# Starch Synthase IIa-Deficient Mutant Rice Line Produces Endosperm Starch With Lower Gelatinization Temperature Than Japonica Rice Cultivars

**DOI:** 10.3389/fpls.2018.00645

**Published:** 2018-05-15

**Authors:** Satoko Miura, Naoko Crofts, Yuhi Saito, Yuko Hosaka, Naoko F. Oitome, Toshiyuki Watanabe, Toshihiro Kumamaru, Naoko Fujita

**Affiliations:** ^1^Department of Biological Production, Faculty of Bioresource Sciences, Akita Prefectural University, Akita City, Japan; ^2^Rice Research Center, Kameda Seika Co., Ltd., Niigata, Japan; ^3^Plant Genetic Resources, Institute of Genetic Resources, Faculty of Agriculture, Kyushu University, Fukuoka, Japan

**Keywords:** amylopectin, amylose, endosperm starch, low gelatinization temperature, protein complex, rice, starch synthase IIa

## Abstract

The gelatinization temperature of endosperm starch in most japonica rice cultivars is significantly lower than that in most indica rice cultivars. This is because three single nucleotide polymorphisms in the *Starch synthase* (*SS*) *IIa* gene in japonica rice cultivars (*SSIIa^J^*) significantly reduce SSIIa activity, resulting in an increase in amylopectin short chains with degree of polymerization (DP) ≤ 12 compared to indica rice cultivars (*SSIIa^I^*). SSIIa forms a trimeric complex with SSI and starch branching enzyme (BE) IIb in maize and japonica rice, which is likely important for the biosynthesis of short and intermediate amylopectin chains (DP ≤ 24) within the amylopectin cluster. It was unknown whether the complete absence of SSIIa further increases amylopectin short chains and reduces gelatinization temperature and/or forms altered protein complexes due to the lack of a suitable mutant. Here, we identify the SSIIa-deficient mutant rice line *EM204* (*ss2a*) from a screen of ca. 1,500 plants of the rice cultivar Kinmaze (japonica) that were subjected to *N*-methyl-*N*-nitrosourea mutagenesis. The *SSIIa* gene in *EM204* was mutated at the boundary between intron 5 and exon 6, which generated a guanine to adenine mutation and resulted in deletion of exon 6 in the mRNA transcript. SSIIa activity and SSIIa protein in developing endosperm of *EM204* were not detected by native-PAGE/SS activity staining and native-PAGE/immunoblotting, respectively. SSIIa protein was completely absent in mature seeds. Gel filtration chromatography of soluble protein extracted from developing seeds showed that the SSI elution pattern in *EM204* was altered and more SSI was eluted around 300 kDa, which corresponds with the molecular weight of trimeric complexes in wild type. The apparent amylose content of *EM204* rice grains was higher than that in its parent Kinmaze. *EM204* also had higher content of amylopectin short chains (DP ≤ 12) than Kinmaze, which reduced the gelatinization temperature of *EM204* starch by 5.6°C compared to Kinmaze. These results indicate that *EM204* starch will be suitable for making foods and food additives that easily gelatinize and slowly retrograde.

## Introduction

Starch synthases (SSs) have a central role in starch biosynthesis. SSs elongate α-1,4-linked linear glucan chains of starch by transferring an ADP glucose (ADPG) residue to the non-reducing end of glucans. SSIIa functions to elongate amylopectin short chains with degree of polymerization (DP) ≤12 to 13 ≤ DP ≤ 24, and is highly conserved in rice (Umemoto et al., 2002; Nakamura et al., 2005), maize (Zhang et al., 2004; Liu et al., 2012), wheat (Yamamori et al., 2000), barley (Morell et al., 2003), sweet potato (Katayama et al., 2002; Kitahara et al., 2005), and Arabidopsis (Zhang et al., 2008).

The rice *SSIIa* gene controls the chain-length distribution of endosperm amylopectin in indica and japonica rice cultivars and corresponds to the *alk* (alkali disintegration of starch granules) gene (Umemoto et al., 1999, 2002). SSIIa from japonica rice cultivars (Nipponbare and Kinmaze) contains four amino acid replacements compared with indica rice cultivars (IR36 and Kasalath), and three of these are associated with significant reduction in japonica SSIIa activity (Nakamura et al., 2005). The SSIIa activity of recombinant SSIIa^J^ derived from the japonica rice cultivars Nipponbare and Kinmaze is estimated as ca. 10% of that of recombinant SSIIa^I^ derived from the indica rice cultivar IR36 (Nakamura et al., 2005). Differences in the amylopectin chain-length distribution significantly affect the starch gelatinization temperature. The gelatinization temperature of japonica rice starch is ca. 8°C lower than that of indica rice starch (Nakamura et al., 2002). This is because long parallel chains of amylopectin in indica rice expressing SSIIa^I^ can form a longer double helix; by contrast, short chains with DP ≤ 12 can only form a short double helix in japonica rice expressing SSIIa^J^.

Recently, direct evidence of protein–protein interactions among starch biosynthetic enzymes was observed in developing endosperms of wheat (Tetlow et al., 2004) and other cereals (Hennen-Bierwagen et al., 2008; Tetlow et al., 2008; Ahmed et al., 2015; Crofts et al., 2015). It is thought that SSIIa forms trimeric complexes with SSI and branching enzyme IIb (BEIIb) in developing endosperms of maize and rice, and these complexes seem to be important for the biosynthesis of short and intermediate starch chains (DP ≤ 24) within the amylopectin cluster (Liu et al., 2012; Crofts et al., 2015, 2017a). It remains to be determined how these trimeric complexes, higher molecular complexes, and monomers relate to the resulting starch structures. Our double mutant rice lines reveal a relay reaction between BEIIb and SSI, where SSI elongates the branches formed by BEIIb (Abe et al., 2014). SSI and BEIIb associate and synergistically enhance their mutual functions as shown by the *in vitro* analyses of recombinant enzymes (Nakamura et al., 2014). SSIIa can complement a large part of SSI function (Crofts et al., 2017b). These combined results indicate that the SSI, SSIIa, and BEIIb isozymes closely communicate during starch biosynthesis.

SSIIa deficiency in maize (Takeda and Preiss, 1993), wheat (Yamamori et al., 2000), barley (Morell et al., 2003), and Arabidopsis (Zhang et al., 2008) leads to higher amylose contents than in wild types (Fujita and Nakamura, 2012). In rice, the *SSIIa* and granule-bound starch synthase I (*GBSSI*) genes, which primarily synthesize amylose, are closely located on chromosome 6. Therefore, most indica rice cultivars have wild-type *SSIIa^I^* and *GBSSI^I^* alleles. By contrast, most japonica rice cultivars have *SSIIa^J^* and *GBSSI^J^* alleles that generate enzymes with low activities (Sano, 1984; Nakamura et al., 2002). Therefore, the true effects of *SSIIa^J^* on amylose content in japonica rice cultivars remains unknown, although the amylose contents of japonica cultivars are lower than those of indica rice cultivars (Sano, 1984).

Although SSIIa^J^ has low SSIIa activity, mutant rice lines with complete SSIIa knockout have not been isolated. Hence, the effects of SSIIa deficiency on other enzymes involved in starch biosynthesis, protein complex formation, apparent amylose content, starch structure, and starch physicochemical properties are unknown. In this study, we isolated an SSIIa-deficient mutant rice line from a chemically mutagenized japonica population and identified the *SSIIa* gene mutation. We evaluated the following rice lines: #1110-290, Kinmaze, and *EM204*. These lines expressed different *SSIIa* alleles (*SSIIa^I^*, *SSIIa^J^*, and *ss2a*, respectively) and the same *GBSSI* allele derived from japonica cultivars (*GBSSI^J^*). We discuss the effects of three *SSIIa* alleles (*SSIIa^I^*, *SSIIa^J^*, and *ss2a*) with the *GBSSI^J^* allele on starch biosynthesis, protein complex formation, apparent amylose content, amylopectin structure, and starch gelatinization temperature.

## Materials and Methods

### Plant Materials

A total of 1,500 mutant rice lines were generated by treating fertilized egg cells with 1 mM *N*-methyl-*N*-nitrosourea as described previously (Satoh and Omura, 1979). The initial screening was performed by measuring the water absorbance properties of boiled rice to isolate possible candidates for low-calorie rice lines. SSIIa-deficient *EM204* (*ss2a*/*GBSSI^J^*) was identified as one of 12 candidate lines by immunoblotting total proteins extracted from mature seeds using antiserum raised against rice SSIIa.

Kinmaze (the parental line, *SSIIa^J^/GBSSI^J^*), IR36 (indica rice cultivar, *SSIIa^I^/GBSSI^I^*), and #1110-290 [*SSIIa* and *GBSSI* genes derived from indica (IR36) and japonica (Nipponbare) cultivars, respectively (*SSIIa^I^/GBSSI^J^*), Crofts et al., 2017b] were used as control lines (**Table 1**). All rice lines were grown in a paddy field of Akita Prefectural University during the summer months under natural conditions.

**Table 1:** Dehulled grain weight and genotypes of rice lines used in this study.

Line	Genotype	Grain weight (mg)
IR36	*SSIIa^I^/GBSSI^I^*	18.9 ± 0.2^a^ (97)^b^
#1110-290	*SSIIa^I^/GBSSI^J^*	16.8 ± 0.2 (87)
Kinmaze	*SSIIa^J^/GBSSI^J^*	19.4 ± 0.5 (100)
*EM204*	*ss2a/GBSSI^J^*	16.5 ± 0.4^∗^ (85)

*^a^Values shown are means ± SE of 20 seeds. ^b^Values in parentheses are the percentages of the wild type (Kinmaze). ^∗^Significant differences between EM204 and Kinmaze (t-test, P<0.01).*

### Nucleotide Sequencing of *SSIIa* Gene From mRNA and Genomic DNA

Total RNA was extracted from developing endosperm of Kinmaze (*SSIIa^J^/GBSSI^J^*) and *EM204* (*ss2a*/*GBSSI^J^*) using the RNeasy Plant Mini Kit (QIAGEN). A full-length rice *SSIIa* cDNA was amplified using the PrimeScript II High Fidelity One-Step RT-PCR Kit (Takara) and a pair of *SSIIa* gene-specific primers (5′-aagcacgcgcacacactcaa-3′ and 5′-catcctattacgcatgcatgatgc-3′). Two primer pairs (5′-ccacaacgtattcagagatcgga-3′ and 5′-gaggta tcgaaggagttcatccgt-3′) were designed to cover the region of deletion in *EM204* (*ss2a*/*GBSSI^J^*). The genomic DNA fragment was isolated and amplified from Kinmaze (*SSIIa^J^/GBSSI^J^*) and *EM204* (*ss2a*/*GBSSI^J^*) leaves using the DNeasy Plant Mini Kit (QIAGEN), and then the sequence was confirmed using the above gene-specific primers.

### Protein Analyses

For immunoblotting, total proteins from one mature rice seed and three developing rice seeds (with the embryo removed) were extracted with 10 volumes of denaturing extraction buffer [0.125 M Tris–HCl, pH 6.8, 8 M urea, 4% sodium dodecyl sulfate (SDS), 5% β-mercaptoethanol], and then incubated at room temperature for 2 h with rotation according to the method of Crofts et al. (2012). Soluble protein (SP), loosely bound to starch granule protein (LBP), and tightly bound to starch granule protein (TBP) were extracted from eight developing endosperm according to a previously published method (Asai et al., 2014; Crofts et al., 2015). Immunoblotting was performed as described previously (Crofts et al., 2012).

Samples used for native-PAGE/activity staining were extracted from three developing endosperm (10–15 DAF) using 1.5 volumes of buffer (for SSIIa zymogram) or 3 volumes of buffer (for the remaining zymograms) relative to seed fresh weight, and equal amounts of protein were loaded from each line (Fujita et al., 1999). SS activity staining for detecting SSI and SSIIIa was performed using glycogen as primer as described previously (Nishi et al., 2001), except that 0.5 M citrate was added to the reaction mixture. SSIIa activity staining was performed as described previously (Nishi et al., 2001) with the following modifications: 9% acrylamide native-PAGE gel containing 0.05% maize amylopectin as primer was electrophoresed at 8 mA for stacking gel and 15 mA for separation gel. The gel was run for an additional 50 min at 15 mA after the dye front reached the bottom, and then incubated with SS reaction buffer containing 0.5 M citrate adjusted to pH 10 with NaOH. BE activity was assessed using gels containing 0.0001% oyster glycogen (Yamanouchi and Nakamura, 1992). Debranching enzyme (DBE) was assessed as described previously (Fujita et al., 1999).

A 700-mg aliquot of developing seeds was extracted and fractionated by gel filtration chromatography using Superdex 200 resin according to the method of Crofts et al. (2015). Native-PAGE/SS and native-PAGE/BE activity staining was performed as described above. Native-PAGE/immunoblotting of each fraction was performed as described by Crofts et al. (2015).

### Analyses of Starch Structure and Physicochemical Properties

Starch granules were purified from three grams of mature rice endosperm to determine starch structure and physicochemical properties according to the method of Fujita et al. (2003). Endosperm amylopectin chain-length distribution was determined by capillary electrophoresis (P/ACE MDQ Carbohydrate System, AB Sciex). The ratio of amylose to amylopectin was determined by gel filtration chromatography (Toyopearl HW-55S and HW-50S × 3) according to previously published methods (Fujita et al., 2007; Toyosawa et al., 2016). The thermal properties of endosperm starch were determined by differential scanning calorimetry (DSC, Seiko Instrument 6100) and analyzed as described previously (Fujita et al., 2003, 2006).

## Results

### SSIIa Deficiency in Mature Endosperm and Detection of the *SSIIa* SNP in *EM204*

**FIGURE 1:**
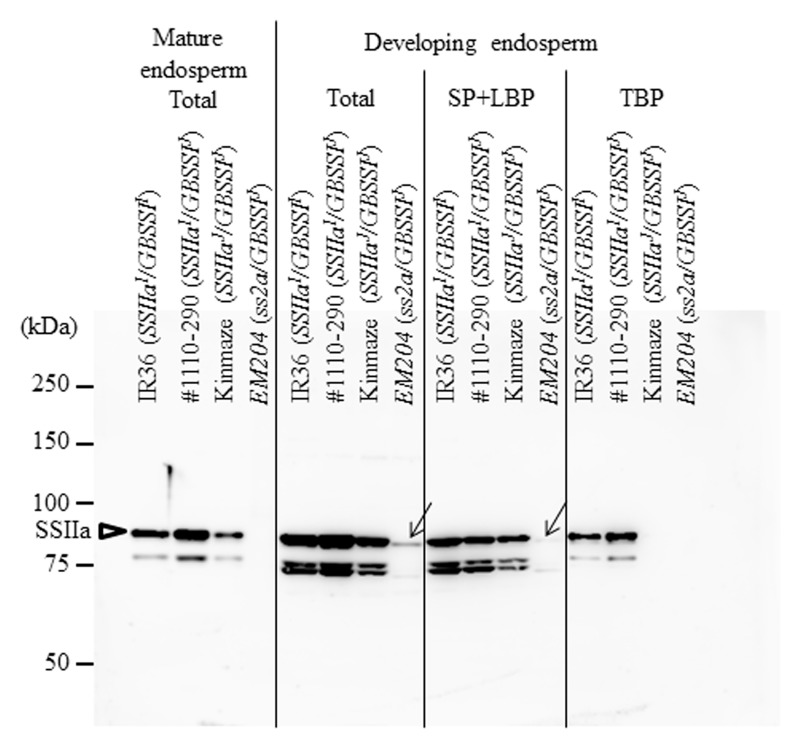
Immunoblotting of mature and developing endosperm using anti-SSIIa serum. Total, total proteins; SP+LBP, soluble and loosely bound protein; TBP, tightly bound protein; IR36, indica rice cultivar, *SSIIa^I^/GBSSI^I^*; #1110-290, *SSIIa^I^/GBSSI^J^*; Kinmaze, japonica rice cultivar, *SSIIa^J^/GBSSI^J^*; *EM204*, *ss2a/GBSSI^J^*. Arrows show the possible truncated SSIIa bands in *EM204*.

**FIGURE 2:**
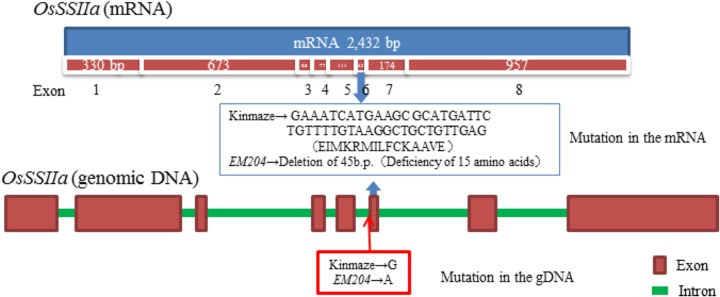
Structure of the *OsSSIIa* gene [mRNA (accession No. AB115916.1 and genomic DNA (accession No. AP003509.3)] and identification of the mutation site in *EM204*.

The SSIIa band (ca. 85 kDa) was not detected in the mature endosperm of *EM204* (*ss2a/GBSSI^J^*) using immunoblotting with a polyclonal antiserum of SSIIa, although it was detected in Kinmaze (*SSIIa^J^/GBSSI^J^*) (**Figure 1**, Mature endosperm, Total). By contrast, weak SSIIa bands with slightly smaller molecular weights were detected in the total protein fraction and in the SP+LBP fraction from *EM204* (*ss2a/GBSSI^J^*) developing endosperm (**Figure 1**, Developing endosperm, Total and SP+LBP). The western blot signals located around 75 kDa were likely to be truncated SSIIa since they were absent in *EM204* (*ss2a/GBSSI^J^*). To identify the mutation in the *SSIIa* gene in *EM204* (*ss2a/GBSSI^J^*), we compared cDNA sequences of *SSIIa* genes from developing endosperm in *EM204* (*ss2a/GBSSI^J^*) and Kinmaze (*SSIIa^J^/GBSSI^J^*). Exon 6 containing 45 base pairs was absent in cDNA of *EM204* (*ss2a/GBSSI^J^*) (**Figure 2**). DNA sequences around exon 6 of *EM204* (*ss2a/GBSSI^J^*) were determined using genomic DNA from young leaves (**Figure 2**). The last nucleotide in the *SSIIa* intron 5 in *EM204* (*ss2a/GBSSI^J^*) was mutated from guanine to adenine (**Figure 2**). This nucleotide substitution resulted in the splicing out of exon 6 together with introns 5 and 6, and led to the deletion of 15 amino acids in SSIIa of *EM204* (*ss2a/GBSSI^J^*; **Figure 2**). The slightly smaller SSIIa detected in developing *EM204* endosperm (*ss2a/GBSSI^J^*; **Figure 1**) might correspond to the SSIIa polypeptide with 15 amino acids deleted (from exon 6), although the amino acid sequence of truncated SSIIa in *EM204* (*ss2a/GBSSI^J^*) was not confirmed. Stereo crystallography from homology modeling of SSIIa in *EM204* (*ss2a/GBSSI^J^*) indicated that the 15 amino acids deleted from SSIIa in *EM204* (*ss2a/GBSSI^J^*) correspond to an α-helix in the N domain close to the catalytic site (**Supplementary Figure S1**). The mutated SSIIa in *EM204* (*ss2a/GBSSI^J^*) may not be enzymatically active or may be more rapidly degraded during endosperm development.

**FIGURE 3:**
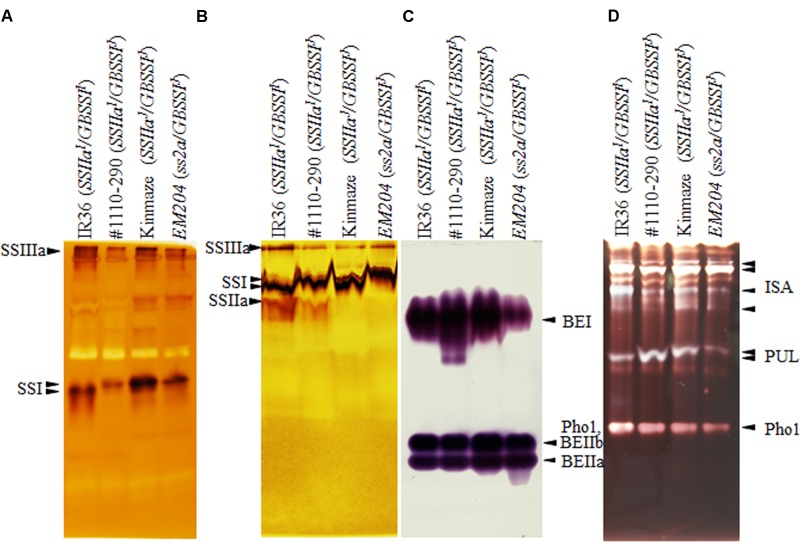
Pleiotropic effects on starch biosynthetic enzymes revealed by native-PAGE/activity staining. **(A)** Starch synthase (SS) I and SSIIIa activity was detected using glycogen as primer. It is noted that SSI derived from Indica rice migrate faster than that of japonica rice (Chen and Bao, 2016; Itoh et al., 2017). **(B)** SSIIa activity was detected using maize amylopectin as primer; reaction was performed at pH 10 to minimize hydrolase activity (see Section “Materials and Methods”). **(C)** Branching enzyme (BE) activity. BEIIb and Phosphorylase 1 (Pho1) activity bands were overlapped. **(D)** Debranching enzyme (DBE) activity. Each isozyme labeled with arrowheads has been identified with corresponding mutant rice lines. IR36, indica rice cultivar, *SSIIa^I^/GBSSI^I^*; #1110-290, *SSIIa^I^/GBSSI^J^*; Kinmaze, japonica rice cultivar, *SSIIa^J^/GBSSI^J^*; *EM204*, *ss2a/GBSSI^J^*.

To detect SSIIa activity, we performed native-PAGE/SS activity staining using a maize amylopectin gel (**Figure 3B**). SSIIa activity bands were detected below the SSI activity band in IR36 (*SSIIa^I^/GBSSI^I^*) and #1110-290 (*SSIIa^I^/GBSSI^J^*), which contain the *SSIIa^I^* allele. By contrast, SSIIa activity bands were not detected in Kinmaze (*SSIIa^J^/GBSSI^J^*) and *EM204* (*ss2a/GBSSI^J^*). A previous *in vitro* study using recombinant SSIIa enzymes estimated that the activity of Kinmaze SSIIa^J^ was 10% of that of SSIIa^I^ (Nakamura et al., 2005). The SSIIa activity of Kinmaze (*SSIIa^J^/GBSSI^J^*) was not detectable on native-PAGE/SS activity staining gels due to the detection limit of this method.

### Pleiotropic Effects of SSIIa Loss on Other Starch Biosynthetic Enzymes

To investigate the effects of loss of SSIIa on starch biosynthetic enzymes, SSs, BEs, DBEs, and plastidial starch phosphorylase (Pho1) were detected by native-PAGE/activity staining (**Figure 3**). Although the zymogram signals are not quantitative, the following pleiotropic effects were observed at least three times (replicated data were not shown). SSI and SSIIIa activity bands were slightly weaker in #1110-290 (*SSIIa^I^/GBSSI^J^*) than in the other lines (**Figure 3A**). The BEI activities in *EM204* (*ss2a/GBSSI^J^*) and #1110-290 (*SSIIa^I^/GBSSI^J^*) were weaker and stronger than those in Kinmaze (*SSIIa^J^/GBSSI^J^*) and IR36 (*SSIIa^I^/GBSSI^I^*), respectively. BEIIa activities were comparable in the four lines (**Figure 3C**). Pho1 activity was weaker in *EM204* (*ss2a/GBSSI^J^*) than in the other lines (**Figures 3C,D**). Isoamylase (ISA) and pullulanase (PUL) activities were slightly weaker in *EM204* (*ss2a/GBSSI^J^*) than in the other lines (**Figure 3D**). PUL activities (**Figure 3D**) were weaker and stronger in IR36 (*SSIIa^I^/GBSSI^I^*) and #1110-290 (*SSIIa^I^/GBSSI^J^*), respectively, than in Kinmaze (*SSIIa^J^/GBSSI^J^*).

**FIGURE 4:**
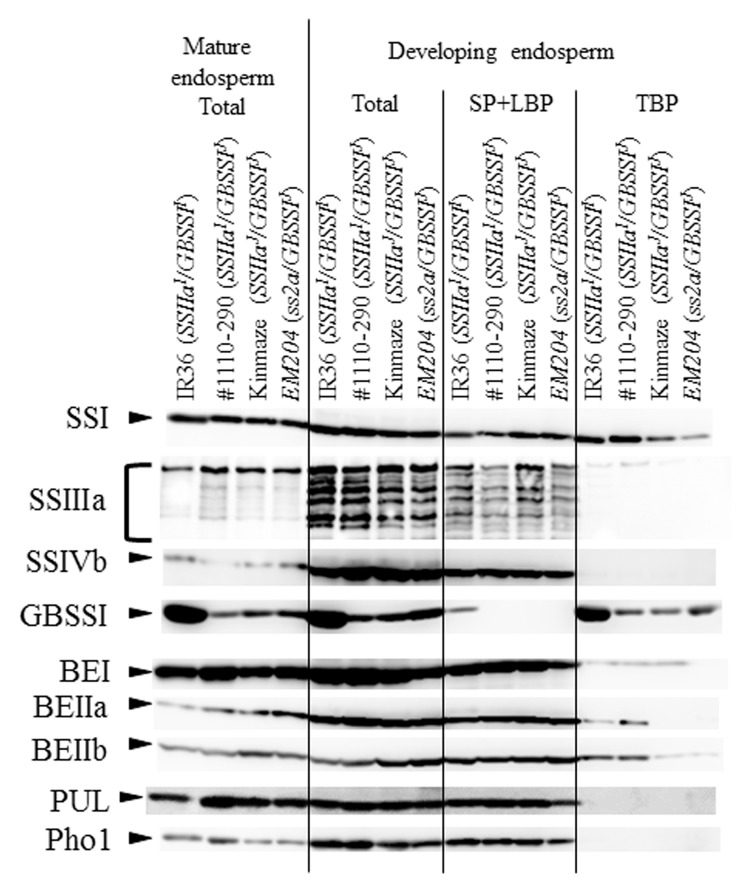
Isozyme distribution in protein fractions [Total protein (Total), soluble protein (SP), loosely bound protein (LBP), and tightly bound protein (TBP)] extracted from developing endosperm (10–15 DAF). Immunoblotting of each fraction was performed using anti-SSI, SSIIa, SSIIIa, SSIVb, GBSSI, BEI, BEIIa, and BEIIb antibodies. IR36, indica rice cultivar, *SSIIa^I^/GBSSI^I^*; #1110-290, *SSIIa^I^/GBSSI^J^*; Kinmaze, japonica rice cultivar, *SSIIa^J^/GBSSI^J^*; *EM204*, *ss2a/GBSSI^J^*.

Total protein, SP+LBP, and TBP extracted from developing endosperm, and total protein extract of mature endosperm were analyzed to detect major starch biosynthetic enzymes by immunoblotting with polyclonal antisera (**Figure 4**). Most of the enzyme levels were similar in total protein extracts of mature endosperm from all lines, except that the GBSSI protein level was much higher in IR36 (*SSIIa^I^/GBSSI^I^*) than in other lines. BEI, PUL, and Pho1 protein levels were higher in #1110-290 (*SSIIa^I^/GBSSI^J^*) than in other lines (**Figure 4**, Mature endosperm, Total). In developing endosperms, SSI protein levels were lower in the TBP fractions from *EM204* (*ss2a/GBSSI^J^*) than in fractions from other lines (**Figure 4**, Developing endosperm, TBP). SSIIa protein bands were not detected in the TBP fractions from Kinmaze (*SSIIa^J^/GBSSI^J^*) and *EM204* (*ss2a/GBSSI^J^*) (**Figure 1**). SSIIIa protein band was not detected in the TBP fractions from *EM204* (*ss2a/GBSSI^J^*), whereas faint SSIIIa bands were detected in IR36 (*SSIIa^I^/GBSSI^I^*), #1110-290 (*SSIIa^I^/GBSSI^J^*), and Kinmaze (*SSIIa^J^/GBSSI^J^*) (**Figure 4**, Developing endosperm, TBP). Total SSIVb levels were lower in IR36 (*SSIIa^I^/GBSSI^I^*) than in other lines. GBSSI levels were much higher in total and TBP fractions from IR36 (*SSIIa^I^/GBSSI^I^*) than in those from other lines and were detected even in the SP+LBP fractions (**Figure 4**, Developing endosperm). GBSSI levels were slightly higher in TBP fractions from *EM204* (*ss2a/GBSSI^J^*) than #1110-290 (*SSIIa^I^/GBSSI^J^*) and Kinmaze (*SSIIa^J^/GBSSI^J^*). BEI levels were lower in total and SP+LBP fractions from *EM204* (*ss2a/GBSSI^J^*) than in those from other lines and were not detected in the TBP fraction. BEIIa protein bands were not detected in the TBP fraction from Kinmaze (*SSIIa^J^/GBSSI^J^*) and *EM204* (*ss2a/GBSSI^J^*). BEIIb levels were lower in the TBP fractions from Kinmaze (*SSIIa^J^/GBSSI^J^*) and *EM204* (*ss2a/GBSSI^J^*) than in those from IR36 (*SSIIa^I^/GBSSI^I^*) and #1110-290 (*SSIIa^I^/GBSSI^J^*) (**Figure 4**, Developing endosperm, TBP). Differences between SSIIa^I^ and SSIIa^J^ binding to starch granules (**Figure 1**, Developing endosperm, TBP) were consistent with previous reports (Umemoto et al., 2004; Nakamura et al., 2005).

### Effects of SSIIa Loss on Starch Biosynthetic Enzyme Complexes in Developing Seeds

**FIGURE 5:**
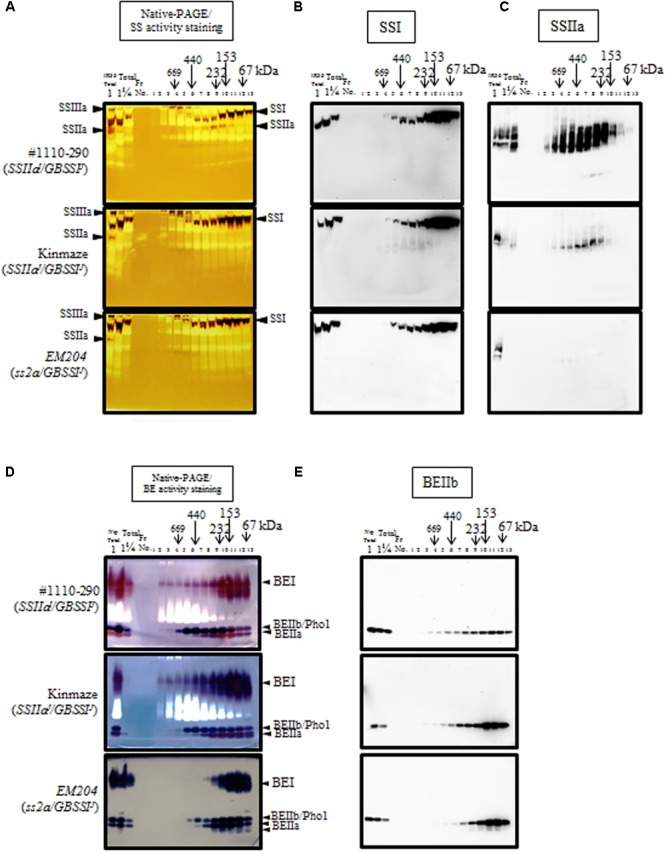
Molecular weight distribution of starch synthase (SS) **(A–C)** and branching enzyme (BE) **(D,E)** analyzed by native-PAGE/activity staining **(A,D)** and immunoblotting of the corresponding gel **(B,C,E)** loaded with the fractions obtained from gel filtration chromatography of soluble extract from rice developing endosperm. #1110-290, *SSIIa^I^/GBSSI^J^*; Kinmaze and Nipponbare, japonica rice cultivar, *SSIIa^J^/GBSSI^J^*; *EM204*, *ss2a/GBSSI^J^*. The left lane of each native-PAGE/activity staining gel and immunoblot is the loading control (IR36 for SS and Nipponbare for BE), except for D and E in Kinmaze, which are blanks. Total is the crude extract before gel filtration chromatography loaded with different amounts of extract (1 and ¼) for semi-quantitative comparisons. More mobile purple bands in *EM204* of BE activity staining **(D)** are also BEIIa.

Soluble extracts from developing seeds containing SSIIa^I^ (#1110-290), SSIIa^J^ (Kinmaze), and *ss2a* (*EM204*) with GBSSI^J^ were fractionated by performing gel filtration chromatography with Superdex 200 resin. The fractions were analyzed by native-PAGE/SS and BE activity staining and immunoblotting using the corresponding native-PAGE gels (**Figure 5**). SSI and SSIIIa activity bands were found in Fractions (Fr.) 5–12 and Fr. 2–6, respectively (**Figure 5A**). SSIIa activity band was detected in. Fr. 8–11 of #1110-290 (*SSIIa^I^/GBSSI^J^*), but not in Kinmaze (*SSIIa^J^/GBSSI^J^*) or *EM204* (*ss2a/GBSSI^J^*) (**Figure 5A**), confirming the native-PAGE/SS activity staining of crude extract result (**Figure 3B**). Immunoblotting of corresponding native-PAGE gels showed that activity bands and immunoblotting signals merged for SSI (**Figure 5B**) and SSIIIa (data not shown). However, immunoblotting signals of SSIIa in #1110-290 (*SSIIa^I^/GBSSI^J^*) were detected in broader area than its activity bands (**Figure 5C**). There are two possible reasons why SSIIa activity was not detected in the faster-migrating area. The first possibility is that some SSIIa proteins happen to co-migrate with glucan hydrolase or transferase on native-PAGE; hence, the glucans generated by SSIIa may be degraded. The second possibility is that some SSIIa proteins became slightly degraded during the experimental procedure, and these inactive proteins likely migrated faster on the native-PAGE gel. The SSIIa activity band was not detected in Kinmaze (*SSIIa^J^/GBSSI^J^*), but SSIIa protein was detected by immunoblotting in Kinmaze (*SSIIa^J^/GBSSI^J^*), whereas neither the activity band nor the immunoblot signal of SSIIa was detected in *EM204* (*ss2a/GBSSI^J^*) (**Figure 5C**). Although some portion of SSI eluted in Fr. 6–9 (<230 kDa) in #1110-290 (*SSIIa^I^/GBSSI^J^*) and Kinmaze (*SSIIa^J^/GBSSI^J^*), the majority of SSI eluted in Fr. 10–13 based on immunoblots. By contrast, more SSI immunoblotting signals in *EM204* (*ss2a/GBSSI^J^*) occurred in Fr. 6–9, with a lower content in Fr. 10–13.

Native-PAGE/BE activity staining (**Figure 5D**) showed that less BEI activity eluted in high molecular weight fractions in *EM204* (*ss2a/GBSSI^J^*; Fr. 2–10) than in #1110-290 (*SSIIa^I^/GBSSI^J^*) and Kinmaze (*SSIIa^J^/GBSSI^J^*). BEIIb immunoblotting showed that BEIIb protein eluted in wide molecular weight range (Fr. 3–13) in #1110-290. By contrast, the majority of BEIIb eluted in Fr. 10–13 in Kinmaze (*SSIIa^J^/GBSSI^J^*) and *EM204* (*ss2a/GBSSI^J^*), and BEIIb was not detected in Fr. >6 in *EM204* (*ss2a/GBSSI^J^*) (**Figure 5D**). The elution patterns of BEIIa, ISA, PUL, and Pho1 were essentially the same in #1110-290 (*SSIIa^I^/GBSSI^J^*), Kinmaze (*SSIIa^J^/GBSSI^J^*), and *EM204* (*ss2a/GBSSI^J^*) (data not shown).

### Seed Morphology and Grain Weight

**FIGURE 6:**
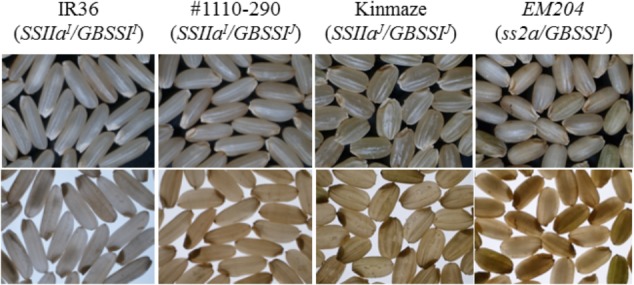
Morphology of rice grains observed using a stereo-microscope with overhead light (upper panels) and on a light box (lower panels). The parts that are chalky or opaque in the seeds appeared dark on a light box. Note that central region of some of *EM204* seeds were chalky. IR36, indica rice cultivar, *SSIIa^I^/GBSSI^I^*; #1110-290, *SSIIa^I^/GBSSI^J^*; Kinmaze, japonica rice cultivar, *SSIIa^J^/GBSSI^J^*; *EM204*, *ss2a/GBSSI^J^*.

Seeds of Kinmaze (*SSIIa^J^/GBSSI^J^*), IR36 (*SSIIa^I^/GBSSI^I^*), and #1110-290 (*SSIIa^I^/GBSSI^J^*) were translucent, whereas some of *EM204* (*ss2a/GBSSI^J^*) seeds were slightly chalky which was observed as dark shadows on a light box (**Figure 6**). The average seed weight (**Table 1**) of *EM204* (*ss2a/GBSSI^J^*; 16.5 mg) was only ca. 85% of that of Kinmaze (*SSIIa^J^/GBSSI^J^*). IR36 (*SSIIa^I^/GBSSI^I^*) seeds had elongated shapes, and the seed weight (18.9 mg) was lower than that of Kinmaze (*SSIIa^J^/GBSSI^J^*). The #1110-290 (*SSIIa^I^/GBSSI^J^*) seeds were shorter than those of IR36 (*SSIIa^I^/GBSSI^I^*) but longer than those of Kinmaze (*SSIIa^J^/GBSSI^J^*), and the average seed weight was 16.8 mg.

### Analysis of Starch Structure and Physicochemical Properties

Apparent amylose content and the ratios of amylopectin short chains and long chains (Fr. III/II) were measured by gel filtration of debranched starch from four rice lines with Toyopearl HW-55S and HW-50S × 3 (**Table 2**). Apparent amylose content was the highest (25.6%) in IR36 (*SSIIa^I^/GBSSI^I^*) among the four rice lines, whereas apparent amylose content of #1110-290 (*SSIIa^I^/GBSSI^J^*) was 13.7% and much lower than that of Kinmaze (20.7%, *SSIIa^J^/GBSSI^J^*). Apparent amylose content of *EM204* (*ss2a/GBSSI^J^*) was 24.1%, significantly higher than that of Kinmaze (*SSIIa^J^/GBSSI^J^*) (**Table 2**). Fr. III/II of IR36 (*SSIIa^I^/GBSSI^I^*; 2.8) and #1110-290 (*SSIIa^I^/GBSSI^J^*; 2.9) was lower than that of Kinmaze (*SSIIa^J^/GBSSI^J^*; 3.2), but there were no significant differences between Kinmaze (*SSIIa^J^/GBSSI^J^*; 3.2) and *EM204* (*ss2a/GBSSI^J^*; 3.3) (**Table 2**).

**Table 2:** Carbohydrate content (weight %) in endosperm starch fractions separated by gel filtration chromatography (Toyopearl HW55S/HW50S x 3).

Line	Genotype	Fr. I^a^	Fr. II	Fr. III	III/II
IR36	*SSIIa^I^/GBSSI^I^*	25.6 ± 0.5**	19.8 ± 0.4	54.6 ± 0.3	2.8 ± 0.1*
#1110-290	*SSIIa^I^/GBSSI^J^*	13.7 ± 0.3**	21.9 ± 0.2	64.4 ± 0.2	2.9 ± 0.0*
Kinmaze^b^	*SSIIa^J^/GBSSI^J^*	20.7 ± 0.2	18.9 ± 0.2	60.4 ± 0.2	3.2 ± 0.1
*EM 204*	*ss2a/GBSSI^J^*	24.1 ± 0.5**	17.8 ± 0.2	58.1 ± 0.7	3.3 ± 0.1

*Mean value ± SE of at least three replications. Every rice line used in this experiment was harvested in 2015. ^a^Three fractions (Fr. I, II, III) were separated at the minima of the carbohydrate content curve detected with refractive index detectors. Fr. I, II, and III correspond to the apparent amylose content, amylopectin long chains, and amylopectin short chains, respectively. ^b^Data from Itoh et al. (2017). ^∗^Significant differences between Kinmaze and other lines (t-test, P<0.05). ^∗∗^Significant differences between Kinmaze and other lines (t-test, P<0.01).*

**FIGURE 7:**
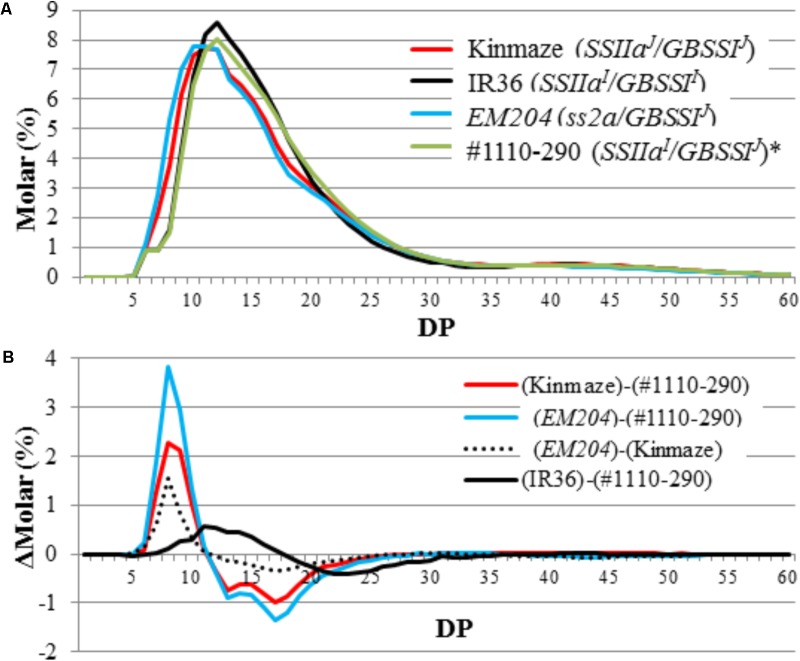
Capillary electrophoresis analysis of amylopectin molecular structure. **(A)** Amylopectin chain-length distribution patterns in mature endosperm. **(B)** Differential plots of Kinmaze (*SSIIa^J^*), *EM204* (*ss2a*), and #1110-290 (*SSIIa^I^*) expressing japonica-type GBSSI. Each panel shows one typical representative data set of at least three replications. DP, degree of polymerization. ^∗^Data from Crofts et al. (2017b).

Chain-length distribution of endosperm amylopectin was analyzed by capillary electrophoresis (**Figure 7**). The contents of short chains with DP = 6–12 and middle chains with DP = 13–24 from Kinmaze expressing SSIIa^J^ were greater and fewer, respectively, than those from IR36 expressing *SSIIa^I^* (Umemoto et al., 1999; Nakamura et al., 2005). The chain-length distributions in #1110-290 expressing SSIIa^I^ showed similar patterns to those in IR36 (**Figure 7A**). By contrast, the contents of short chains with DP = 6–12 and middle chains with DP = 13–24 from *EM204* (*ss2a/GBSSI^J^*) were greater and fewer, respectively, than those from Kinmaze (*SSIIa^J^/GBSSI^J^*) (**Figure 7B**). These results suggest that the function of SSIIa in *EM204* (*ss2a/GBSSI^J^*) was completely impaired or at least lower than that in Kinmaze (*SSIIa^J^/GBSSI^J^*), whose recombinant SSIIa activity was estimated as only ca. 10% of that in IR36 (*SSIIa^I^/GBSSI^I^*) *in vitro* (Nakamura et al., 2005).

The gelatinization temperature of endosperm starch from japonica rice cultivars is much lower than that of endosperm starch from indica rice cultivars (Nakamura et al., 2002). The onset, peak, and conclusion temperatures of Kinmaze (*SSIIa^J^/GBSSI^J^*), endosperm starch were 16.5°C, 13.5°C, and 10.8°C, respectively, lower than those of IR36 (*SSIIa^I^/GBSSI^I^*) (**Table 3**). Those of *EM204* (*ss2a/GBSSI^J^*) were 11.5°C, 5.6°C, and 3.1°C lower than those of Kinmaze (*SSIIa^J^/GBSSI^J^*). By contrast, those of #1110-290 (*SSIIa^I^/GBSSI^J^*) were 6.2°C, 5.1°C, and 5.1°C higher than those of IR36 (*SSIIa^I^/GBSSI^I^*).

**Table 3:** Thermal properties of endosperm starch as determined by differential scanning calorimetry.

Line	Genotype	T_o_^a^ (°C)	T_p_^b^ (°C)	T_c_^c^ (°C)	ΔH^d^ (mJ/mg)
IR36	*SSIIa^I^/GBSSI^I^*	61.9 ± 0.1	66.4 ± 0.1*	70.4 ± 0.0*	14.3 ± 0.5*
#1110-290	*SSIIa^I^/GBSSI^J^*	68.1 ± 0.1*	71.5 ± 0.0*	75.5 ± 0.1*	16.4 ± 0.8*
Kinmaze	*SSIIa^J^/GBSSI^J^*	45.4 ± 0.2	52.8 ± 0.0	59.6 ± 0.2	9.5 ± 0.4
*EM204*	*ss2a/GBSSI^J^*	33.9 ± 0.6*	47.2 ± 0.4*	56.5 ± 0.2*	7.1 ± 0.0*

*Mean value ± SE of three seeds.*

*^a^Onset temperature, ^b^Peak temperature, ^c^Conclusion temperature, ^d^Gelatinization enthalpy of starch. ^∗^Significant differences between Kinmaze and other lines (t-test, P<0.01).*

## Discussion

### Isolation of SSIIa-Deficient Mutant Rice Line

A mutant rice line lacking SSIIa protein in mature seeds was isolated in this study and designated as *EM204* (*ss2a/GBSSI^J^*). Chain-length distribution analysis of endosperm starch showed that the amount of amylopectin short chains with DP = 6–12 was larger in *EM204* (*ss2a/GBSSI^J^*) than in the parental Kinmaze (*SSIIa^J^/GBSSI^J^*), a WT japonica rice cultivar (**Figure 7B**). This indicates that SSIIa in japonica rice cultivars slightly elongates DP = 6–12 amylopectin chains to DP = 13–24 *in vivo*. SSIIa activity bands were not detected in *EM204* (*ss2a/GBSSI^J^*) (**Figures 3B**, **5**), and SSIIa protein was not detected in any gel filtration chromatography fractions from developing endosperm (**Figure 5C**). Although it is possible that truncated SSIIa in *EM204* (*ss2a/GBSSI^J^*) can be translated, the truncated SSIIa in developing endosperm is likely easily degraded during seed development, resulting in lower SSIIa activity in *EM204* (*ss2a/GBSSI^J^*) than in Kinmaze (*SSIIa^J^/GBSSI^J^*) based on the enzyme stereography model (**Supplementary Figure S1**).

*EM204* (*ss2a/GBSSI^J^*) was initially screened as one of 12 mutant lines for low-calorie rice with high water absorption in boiled polished rice (see Materials and methods). The water content in *EM204* (*ss2a/GBSSI^J^*) boiled rice was much higher than that in Kinmaze (*SSIIa^J^/GBSSI^J^*) when polished rice grains were boiled with excess water (data not shown). We expected that the *SSIIa* gene would correspond to a gene involved in water absorption. However, genetic analyses of the F_2_ population resulting from a cross between *EM204* (*ss2a/GBSSI^J^*) and the WT (*SSIIa^J^/GBSSI^J^*) showed that loss of SSIIa and loss of water absorption did not segregate identically. It is possible that gene pathways related to water absorption of polished rice grains consist of multiple genes (data not shown). It will be necessary to use genetic analyses of *EM204* (*ss2a/GBSSI^J^*) to isolate genes related to water absorption other than the *SSIIa* gene.

### Pleiotropic Effects of SSIIa Protein Loss on the Elution Pattern of Starch Biosynthetic Enzymes

Starch biosynthetic enzymes are thought to form protein complexes for efficient starch biosynthesis (Crofts et al., 2017a). Trimeric complexes containing SSIIa, SSI, and BEIIb have been reported in maize (Liu et al., 2012) and japonica rice (Crofts et al., 2015). This trimeric complex is likely involved in generating amylopectin clusters (Crofts et al., 2017a). Therefore, differences in the composition and function of protein complexes modulated by active SSIIa (SSIIa^I^), low-activity SSIIa (SSIIa^J^), or loss of SSIIa (*ss2a*) were suspected. Native-PAGE activity staining and immunoblotting of corresponding gels loaded with the gel filtration chromatography elution fractions suggested that *EM204* (*ss2a*) may form altered trimeric protein complexes compared with those of #1110-290 with active SSIIa (SSIIa^I^) or Kinmaze with low-activity SSIIa (SSIIa^J^). Compared with the SSI elution pattern in #1110-290 (*SSIIa^I^/GBSSI^J^*) and Kinmaze (*SSIIa^J^/GBSSI^J^*), more SSI in *EM204* (*ss2a/GBSSI^J^*) eluted in Fr. 6–9 where the trimeric protein complex elutes than in Fr. 10–13 where monomeric SSI elutes (**Figure 5B**). BEIIb eluted in a wide range of molecular weights in #1110-290 (*SSIIa^I^/GBSSI^J^*). However, the majority of BEIIb in Kinmaze (*SSIIa^J^/GBSSI^J^*) and *EM204* (*ss2a/GBSSI^J^*) was eluted in fractions corresponding to monomeric molecular weight (Fr. 11–13); in *EM204* (*ss2a/GBSSI^J^*), BEIIb was not detected in high molecular weight fractions (<Fr. 5). Based on these results, it can be speculated that SSIIa^J^ in Kinmaze may form alternative protein complex with SSI and BEIIb, whereas the protein complex in *EM204* (*ss2a/GBSSI^J^*) may be generated by BEIIb associating with two SSI proteins but without SSIIa, although the alteration in actual protein complex composition is to be analyzed. The mature seed weight of *EM204* (*ss2a/GBSSI^J^*) was 15% less than its parent Kinmaze (*SSIIa^J^/GBSSI^J^*), indicating that amylopectin synthesis was reduced. Complete absence of SSIIa in *EM204* (*ss2a/GBSSI^J^*) resulted in inefficient elongation of amylopectin branches and altered the elution pattern of starch biosynthetic proteins as analyzed by gel filtration chromatography. It cannot be ruled out that the alteration of elution pattern of proteins through gel-filtration chromatography may be a result of interactions of proteins with truncated SSIIa products. However, it is hard to distinguish them due to the resolution limit of gel filtration chromatography. Further analyses are required to detect the direct evidences of protein–protein interaction such as immune-precipitation and/or the reconstitution experiments using recombinant enzymes to confirm the change in composition of trimeric protein complex.

The *su2* mutants in maize lack SSIIa activity. Some *su2* mutants lack only SSIIa activity and others lack both activity and protein, but all *su2* mutants display increased DP = 6–12 and reduced DP = 13–24 levels in amylopectin chain-length distributions (Zhang et al., 2004), which is the same as that observed in *EM204* (*ss2a/GBSSI^J^*). Protein complex formation in *su2* was analyzed using the *su2* mutant with inactive SSIIa protein, and that trimeric protein complex composition remained the same as the WT (Liu et al., 2012). SSI, SSIIa, and BEIIb in this *su2* mutant displayed a loss of or reduced affinity to the starch granule, which is commonly observed in japonica rice (*SSIIa^J^/GBSSI^J^*) and *EM204* (*ss2a/GBSSI^J^*) and in *ss2a* mutants in wheat (Yamamori et al., 2000) and barley (Morell et al., 2003). Although protein–protein interactions of starch biosynthetic enzymes are likely to be an important mechanism for efficient starch synthesis (Crofts et al., 2017a), the relationship between formation of the SSI-SSIIa-BEIIb trimeric complex, its affinity to the starch granule, and starch structure remains unknown and requires further analyses.

### Effects of *SSIIa* and *GBSSI* Alleles on Starch Structure in Rice Endosperm

Amylose content is higher in the storage starches of *SSIIa*-deficient mutants of maize (Takeda and Preiss, 1993), wheat (Yamamori et al., 2000), barley (Morell et al., 2003), and Arabidopsis (Zhang et al., 2008) than in the corresponding WT plants (Fujita and Nakamura, 2012). Japonica rice cultivars correspond to the *SSIIa* mutant, whereas indica rice cultivars correspond to the WT. However, most japonica cultivars have a leaky mutation in the *GBSSI* gene, which complicates the determination of direct effects of loss of SSIIa on amylose content in rice.

In this study, we compared the amylose contents in three rice lines that expressed a *GBSSI^J^* allele along with three different *SSIIa* alleles: *SSIIa^I^* (#1110-290), *SSIIa^J^* (Kinmaze), and *ss2*a (*EM204*) (**Table 2**). The apparent amylose content of *EM204* (*ss2a/GBSSI^J^*) starch was 3.4% higher than that of the Kinmaze (*SSIIa^J^/GBSSI^J^*), indicating that loss of *SSIIa* in rice leads to increased amylose content as observed in other crops. This is because SSIIa deficiency reduces amylopectin synthesis and GBSSI uses the excess ADPG for amylose synthesis. Moreover, slightly higher expression of *GBSSI* in *EM204* (*ss2a/GBSSI^J^*) than Kinmaze (*SSIIa^J^/GBSSI^J^*) might be another explanation (**Figure 4**). The apparent amylose content of #1110-290 (*SSIIa^I^/GBSSI^J^*), which still has an active SSIIa, was 7% lower than that of Kinmaze (*SSIIa^J^/GBSSI^J^*), which already has significantly reduced SSIIa activity (**Table 2**). The vigorous amylopectin synthesis in #1110-290 (*SSIIa^I^/GBSSI^J^*) expressing an active SSIIa leads to a shortage of ADPG levels in the amyloplast and reduces amylose synthesis. The apparent amylose content of the near-isogenic rice lines (NILs) that have *SSIIa^I^* from indica rice (Kasalath) with Nipponbare (japonica) background (*GBSSI^J^*) was approximately 0.9–2.1% lower than that of Nipponbare (Umemoto et al., 2004). A reduction of 7% of the apparent amylose content in #1110-290 (*SSIIa^I^/GBSSI^J^*) could be due to endosperm development under high temperature because the flowering time of #1110-290 (*SSIIa^I^/GBSSI^J^*) was 3 weeks earlier than that of Kinmaze (*SSIIa^J^/GBSSI^J^*). The average temperature at 20 days after flowering in #1110-290 (*SSIIa^I^/GBSSI^J^*) was 25.1°C, which is 4°C higher than that in Kinmaze (*SSIIa^J^/GBSSI^J^*). The amylose content depends on the temperature during seed development (Hirano and Sano, 1998). An SNP at the splice site in the first intron of *GBSSI* is associated with different temperature sensitivities during seed development (Larkin and Park, 1999). Japonica rice cultivars expressing *GBSSI^J^* accumulate fewer mature *GBSSI* mRNA transcripts at high temperatures than at low temperatures (Zhang et al., 2013).

The apparent amylose content of IR36 (*SSIIa^I^/GBSSI^I^*) with high expression of *GBSSI^I^* was ca. 12% higher than that of #1110-290 (*SSIIa^I^/GBSSI^J^*) with low expression of *GBSSI^J^*, as expected (**Table 2**). By contrast, the amylopectin chain-length distributions were not identical in both lines. The amounts of shorter chains with DP ≤ 17 and middle chains with 19 ≤ DP ≤ 30 in IR36 (*SSIIa^I^/GBSSI^I^*) were higher and lower, respectively, than those of #1110-290 (*SSIIa^I^/GBSSI^J^*) (**Figure 7B**). These changes in amylopectin structure result in a lower starch gelatinization temperature in IR36 (*SSIIa^I^/GBSSI^I^*) than in #1110-290 (*SSIIa^I^/GBSSI^J^*) (**Table 3**). Alternatively, more vigorous SSIIa activity along with low activities of the other SS isozymes [SSI (**Figure 3**) and GBSSI^J^] in #1110-290 (*SSIIa^I^/GBSSI^J^*) may lead to elongation of shorter chains with DP ≤ 17 to 19 ≤ DP ≤ 30.

## Conclusion

In this study, we isolated an *ss2a* rice mutant designated *EM204* (*ss2a/GBSSI^J^*), which completely lacked SSIIa in mature seed and accumulated only very low levels of truncated SSIIa in developing endosperm (**Figure 1**). Analyses of starch biosynthetic protein complex formation in *EM204* (*ss2a/GBSSI^J^*) suggested that loss of SSIIa may be compensated by SSI, which might form an alternative trimeric protein complex (**Figure 5**). *EM204* (*ss2a/GBSSI^J^*) grain accumulated starch with unique properties. The gelatinization temperature (*T*_p_°C) of *EM204* (*ss2a/GBSSI^J^*) endosperm starch was very low, 5.6°C lower than typical japonica rice (**Table 3**). The amylose content was significantly higher in *EM204* (*ss2a/GBSSI^J^*) than in WT (*SSIIa^J^/GBSSI^J^*) (**Table 2**).

The processing methods and retrogradation of starch foods during storage are greatly affected by differences in the starch gelatinization temperature. *EM204* (*ss2a/GBSSI^J^*) starches with low gelatinization temperatures (**Table 3**) could be used for generating foods and additives that retrograde slowly. However, the seed weight of *EM204* (*ss2a/GBSSI^J^*) was 15% lower than that of the WT (*SSIIa^J^/GBSSI^J^*) (**Table 1**). It will be necessary to improve the agricultural traits of *EM204* (*ss2a/GBSSI^J^*) by crossing it with elite cultivars before it can be used for practical applications.

## Author Contributions

SM performed the experiments and prepared the figures and tables. NC performed the experiments and wrote the article. YS performed the experiments. YH and NO provided technical assistance to SM. TW and TK complemented the writing, and NF conceived the original research plans, supervised, and complemented the writing.

## Conflict of Interest Statement

The authors declare that the research was conducted in the absence of any commercial or financial relationships that could be construed as a potential conflict of interest. The reviewer IJT and handling Editor declared their shared affiliation.

## Supplementary Material

FIGURE S1 Homology modeling comparison of the SSIIa stereo structure in Kinmaze (*SSIIa^J^/GBSSI^J^*) and *EM204* (*ss2a/GBSSI^J^*). Images were rendered in Spanner in the Sequence-To-Function Annotation Service. Proteins are represented as a ribbon diagram. N- and C-terminal domains are shown in blue and red, respectively. KTGGL motifs (green) are important for the active site. The yellow loop indicates the 15 amino acids in Kinmaze that were absent in *EM204* (*ss2a/GBSSI^J^*).
