# Supplementary figures and images for: Starch Synthase IIa-Deficient Mutant Rice Line Produces Endosperm Starch With Lower Gelatinization Temperature Than Japonica Rice Cultivars
Source: Front Plant Sci. 2018 May 15;9:645. doi: 10.3389/fpls.2018.00645 (PMC5962810; doi:10.3389/fpls.2018.00645)

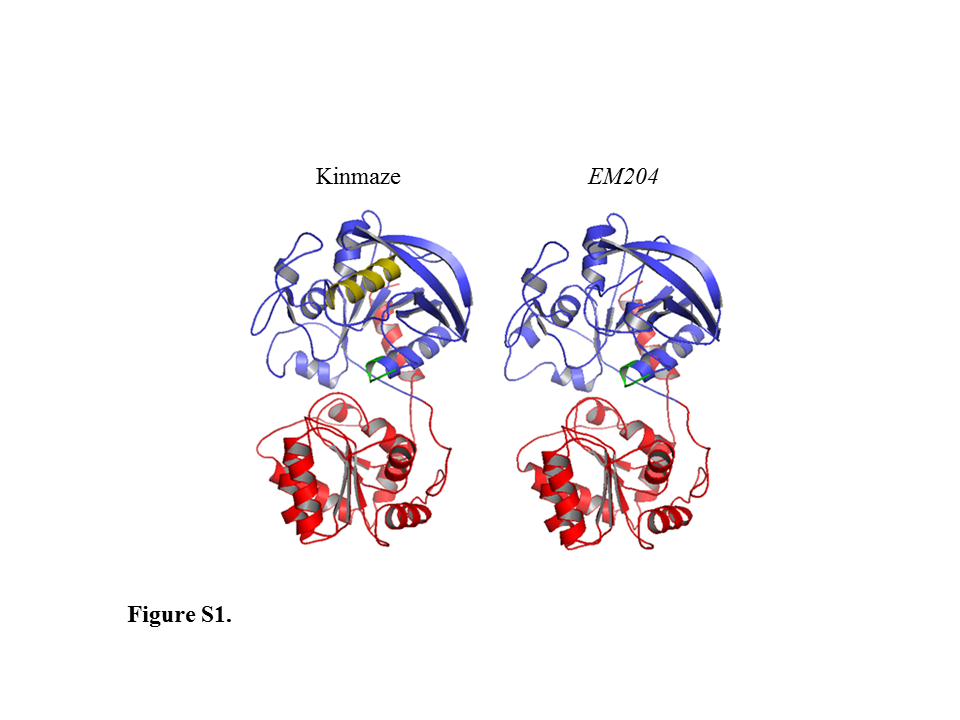

Supplement: FIGURE S1 — Homology modeling comparison of the SSIIa stereo structure in Kinmaze (SSIIaJ/GBSSIJ) and EM204 (ss2a/GBSSIJ). Images were rendered in Spanner in the Sequence-To-Function Annotation Service. Proteins are represented as a ribbon diagram. N- and C-terminal domains are shown in blue and red, respectively. KTGGL motifs (green) are important for the active site. The yellow loop indicates the 15 amino acids in Kinmaze that were absent in EM204 (ss2a/GBSSIJ). [file Image_1.TIF]
